# Sutureless prepuceplasty with wound healing by second intention: An alternative surgical approach in children's phimosis treatment

**DOI:** 10.1186/1471-2490-8-6

**Published:** 2008-03-04

**Authors:** Efstratios Christianakis

**Affiliations:** 1 Department of Pediatric Surgery, Pendeli Children's Hospital, Palaia Pendeli, Athens, Greece

## Abstract

**Background:**

A new technique for the treatment of children's phimosis is presented that minimizes the repairing time, the postoperative complications and maintains the physical foreskin appearance intact.

**Methods:**

Eightyseven children with phimosis were treated with this new developed technique, between 2003 and 2005. Sutureless prepuceplasty creates a permanent surgical extension of the close prepuce. Stretching and retraction of phimotic foreskin reveals a tight prepuce ring that is cutting in its dorsal surface longitudinally. Rarely triple symmetric incisions in the preputial outlet are necessary. The foreskin is loose and moves absolutely free in bilateral courses. The wounds are healing by second intention. Antisepsis, steroids and Elicina cream, (which contains allantoin, collagen, elastin, glycolic acid and vitamins A, D, and E) should apply daily, for twenty to thirty days.

**Results:**

The foreskin is moving in centripetal or efferent courses absolutely loosely, painlessly and bloodlessly. The mean time of follow-up was 27 months (one to four years). No complications were observed.

**Conclusion:**

Sutureless prepuceplasty may present an acceptable alternative in children's phimosis reconstruction.

## Background

Circumcision is probably the oldest and one of the most common elective procedures all over the world. It was initially performed for ritual, religious or family traditional reasons [1]. Simultaneously, circumcision was probably performed for phimosis treatment. Another ancient surgical method for phimosis treatment was the dorsal slit operation, which is a super incision, with no tissue removed [2]. The value of foreskin properties as well as aesthetic reasons determined to other operations preserving the foreskin. The most interesting was the report of prepuceplasty suggested by Homlund DE in 1973. It is a limited dorsal incision of the phimotic prepuce with transverse skin closure with absorbable sutures [3]. Although it is a good operation, it is not without complications or re-operations [4-6]. Sutureless prepuceplasty (SLP) is a faster, easier, painless and without complications technique, which has excellent cosmetic results.

## Methods

Between 2003 and 2005, eightyseven boys (87) with tight phimotic rings, most of whom were resistant to conservative treatment with locally administered steroids for 20 days, were treated with sutureless prepuceplasty (SLP), by the same surgeon). The age of the patients ranged from 2 to 14 years (mean age being 3, 7 years). The problem was that in 27 cases the retractility of the foresking was difficult and in 52 cases impossible. Three operated patients suffered from paraphimoses, while five patients that were treated with the present technique presented recurrent phimosis after partial circumcision (three) and classic sutured prepuceplasty (two).

In eighty one boys a limited dorsal slit was performed , while the remaining six received a triple preputial orifice incision like reverse Mercedes-Benz emblem. Fifty of our patients that received SLP were simultaneously treated for another problem. Twelve of them presented inguinal hernias, ten hydroceles, nine non-retractile testes, three a buried penis, and the remaining sixteen various other surgical problems. Especially in cases of buried penis, where circumcision is contraindicated, SLP was considered as the first choice operation.

Patients with phimotic scars due to balanitis xerotica obliterans were excluded from this study.

All operations were sutureless with wound healing by second intention. All children under the age of 8 were operated on under general anesthesia, while most of the children over 8 years of age (those who were cooperative) were operated upon under local anesthesia with sedation. Postoperative foreskin retraction twice a day for 20 days was mandatory for all patients.

The initial idea of developing the current technique is based on the excellent secondary healing of the prepuce. This is obvious especially in traumas due to edema secondary to nephrotic syndrome, and in particular operations like paraphimoses and hypospadia. In the above mentioned cases where the skin grafts are closed with tension, the longitudinal incision on the stenotic ring or in the dorsal part of the prepuce can solve not only the problem, but also guarantees an excellent healing without the necessity of suturing the trauma.

### Description of the Technique

**Figure 1:**
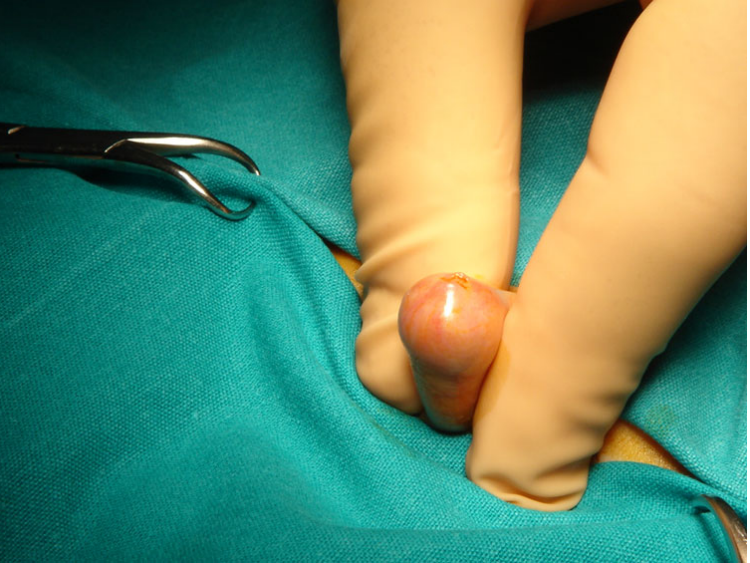
Photo of a young child with severe phimosis. The foreskin cannot be retracted to exposure the glan.

**Figure 2:**
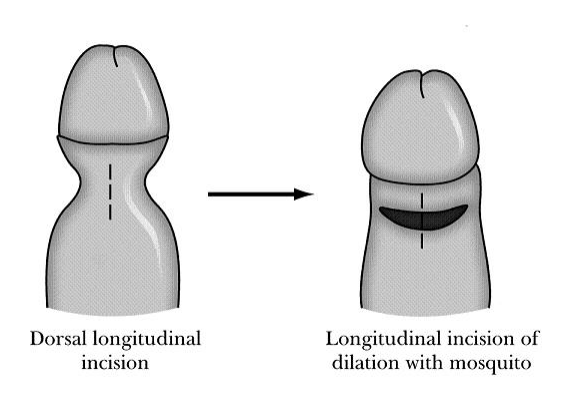
Foreskin is retracted under anesthesia with constriction of the penile shaft forming a clepsydras shape. A longitudinally dorsal incision is made including the skin and dartos. Tissue stretching in transverse direction is performed by using a mosquito forceps, until the clepsydrae ring disappears circumferentially.

**Figure 3:**
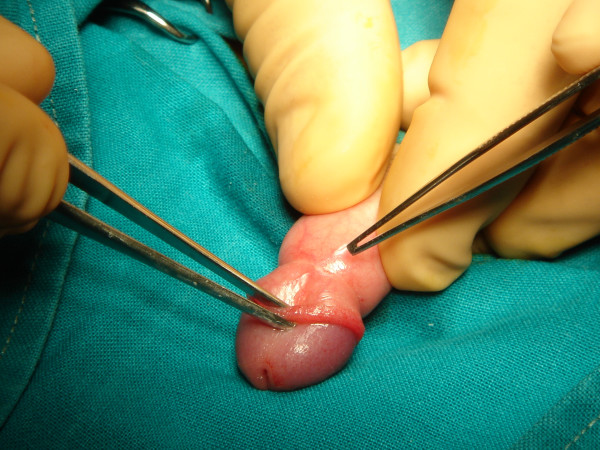
Photo of the stenotic ring of the prepuce.

**Figure 4:**
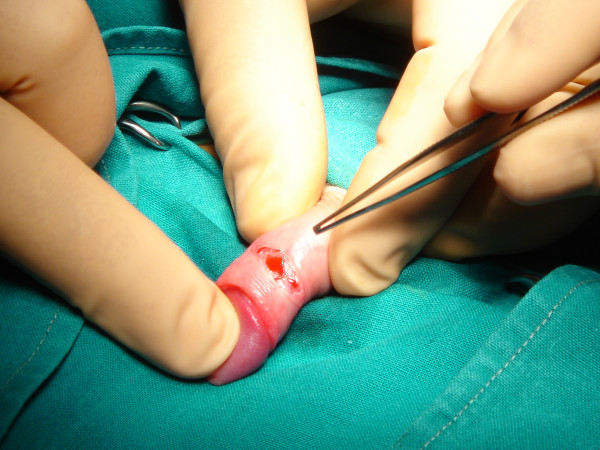
Photo of the longitudinally dorsal incision that is made including the skin and dartos. The dissection and the dilation of the prepuce are performed to ensure looseness of the foreskin during opening and closure.

**Figure 5:**
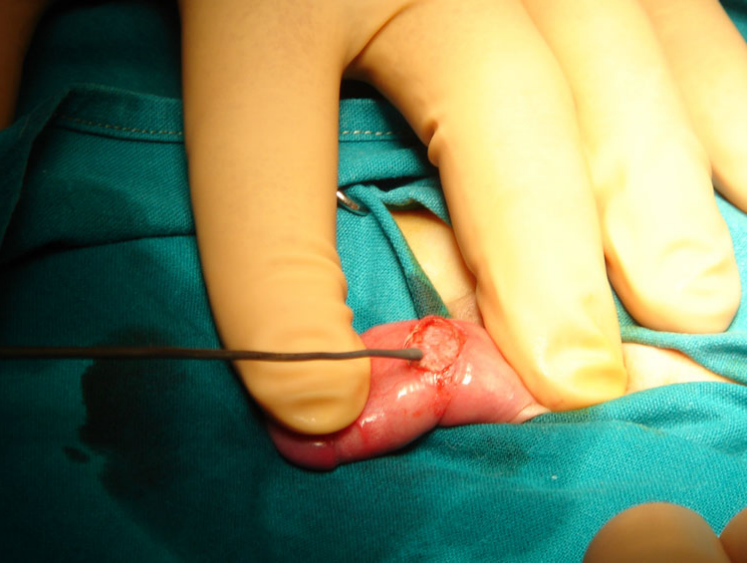
Photo of haemostasis performed with a heatened probe.

The proposed technique can be performed either with local or general anesthesia in patients with phimosis (Figure 1). The anesthetic procedure mainly depends on the concurrent problem of the patient. Local anesthesia is used in selective patients with mild phimosis. A distention of preputial outlet with a mosquito forceps is performed repetitively, and then the foreskin is retracted reversely and if there are preputial adhesions these are separated. An adrenaline-xylocaine 2% solution is injected to the site of the expected dissection to ensure perioperative haemostasis and postoperative analgesia. A dorsal longitudinal incision in the stenotic ring of the prepuce till the cyclic print in the foreskin circumference will disappear (Figures 2, 3). The width of the prepuce stenotic ring varies and may range from 3 to 12 mm including the inner and outer lamina. The medial lamina is usually affected. Pulling the foreskin backwards, you can see a white cyclic banding that causes the malformation of the penis. The underlying dartos is then stretched with a mosquito forceps too, loosening all around tissues until Buck fascia will be visible. The dissection and the dilation of the prepuce are performed to ensure looseness of the foreskin during opening and closure (Figure 4). Haemostasis is performed with a heated probe using the flame of an alcohol lamp or with bipolar electrodiathermy (Figure 5). Finally, the trauma is cleaned with an antiseptic solution of povidone iodine (Betadine) and a cream containing fucidic acid and betamethasone valerate (Betafusine). The trauma is left opened to heal by secondary intention.

**Figure 6:**
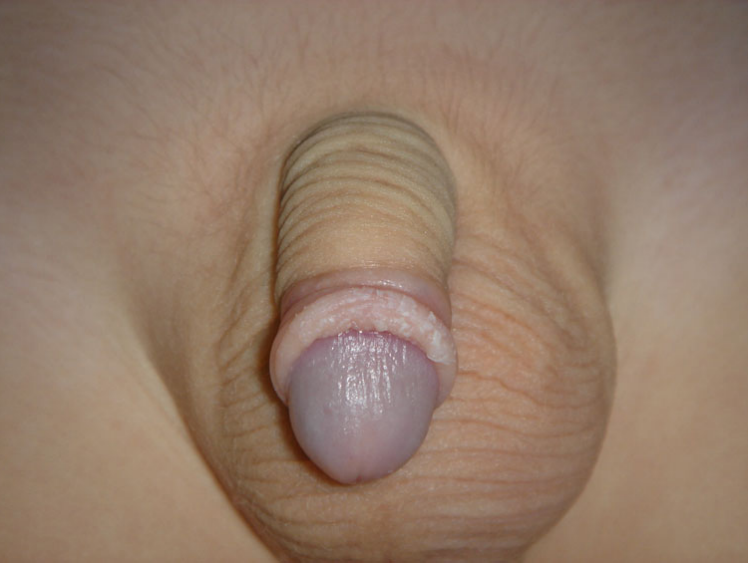
Photo of the final result, six (6) months with the foreskin retracted.

**Figure 7:**
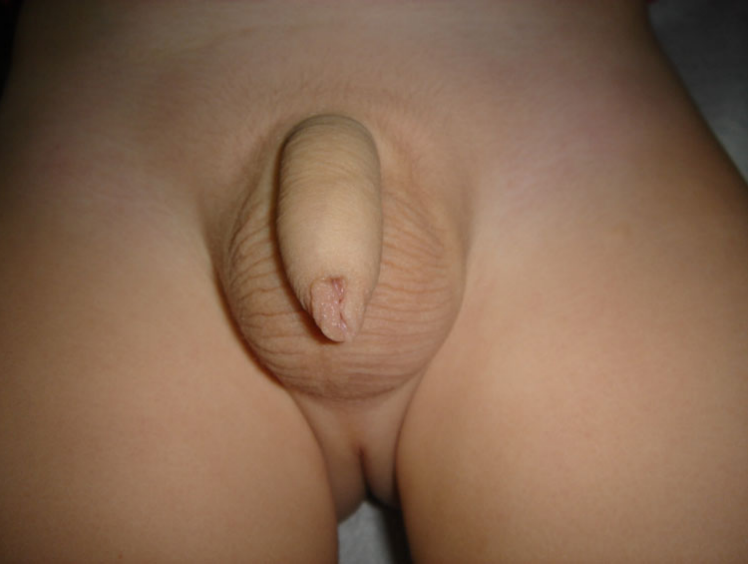
Photo of the final result, six (6) months later with the foreskin closed.

The prepuce should be pulled out daily expositing the glan. Betadine solution (iodine-povidone) is being applied every 12 hours for the next 20 days. After cleaning the trauma with Betadine solution, we place Betafusine cream on it for the first 10 days. From the 11th to 20th postoperative day we cover the trauma with Elicina, which contains allantoin, collagen, elastin, glycolic acid and vitamins A, D, and E, instead of Betafusine. The dilatations of the prepuce should be performed twice a day for at least 20 days. The healing is performed initially following the pattern of the dilated prepuce (and not that of the stenotic). The healed surface looks spidery and wide. On the contrary in the routine technique we are suturing the prepuce transversally and the trauma is primarily closed. If "ear dogs" remain in the trauma after the incision, these should also be incised because they can cause a permanent malformation. The total operative time ranges from 3 to 5 minutes, and the postoperative aesthetic result is excellent (Figures 6, 7).

## Results

Macroscopically the wound healing is completed in 20 days. In younger children, the healing is faster and the postoperative result better. All 87 boys had an excellent healing result, without recurrences or other complications. Postoperatively, 15 patients had a transient subcutaneous trauma edema for 1–2 days. Two of them did not follow the instructions of retracting the foreskin daily for cleaning and four years later developed a mild preputial stenosis, which disappeared within 15 days after topical employment of steroid cream. Indications of SLP are all types of phimosis and especially phimotic buried penis, paraphimoses and operated phimosis recurrences.

## Discussion

In order to maintain the phimotic prepuce intact postoperatively, many authors have suggested their personal techniques. These except for the already mentioned [2-6] are reported as variations of Z-plasty [7], multiple Y-V plasties [8], lateral prepuceplasty [9], multiple internal foreskin lamina [10] and triple incision plasty [11]. The surgeon should select patients with a real problem of phimotic prepuce and not pseudophimosis, which may be solved with topical apposition corticoid cream [12].

There are few in vivo studies evaluating the tissue reaction to suture materials that mainly depend on how the suture polymer interacts with the tissues. Synthetic polyesters, which we usually use in phimosis surgery, degrade with hydrolysis and cause minimal tissue reaction, although they have been associated, in some cases, with recurrences.

On the other side, the healing process is better in growth especially in younger patients. With SLP we interfere with the healing process and differentiate the healing mechanism in wound epithelium formation that we leave the wound to heal "of its own accord", but with daily foreskin retractions. So, finally the widening of the foreskin is permanent.

Sutureless prepuceplasty also possesses significant advantages. It is a faster, bloodless and with lower cost operation (avoiding suturing and shortening the hospitalization), the foreskin retraction is painless, the prepuce is left loose and without tension, there are no suture materials which may induce reactions, there are no postoperative scars or recurrences, the prepuce, postoperatively, looks more natural, the initial results are better and the parents seem to prefer this technique. When phimosis coexists with excessive prepuce, postoperative appearance after prepuceplasty maintains the same. So, the parents have to decide for an operation that may preserve the foreskin intact or not.

The main differences between sutured and sutureless prepuceplasty are the following: After dorsal relieving incision of the phimotic foreskin, it retracts loosely without tension. The stitching of the wound causes a minor or middle grade of difficulty for the same movements. The way of healing is different, without sutures in the second procedure SLP it's **"a moving tissue healing"**. Initially the healing in SLP by second intention is **retarded**, because it is under anti-inflammatory action of topical steroids cream for 10 days, in order to maintain the prepuce loose and prevent the postoperative adhesions' formation. This is followed by a **faster stage **of directed healing, using cicatrizing creams topically.

Finally, the last difference refers to the way of increasing the narrowing prepuce surface. In SLP, we let the wound close in a functional way, leading to the desiring result. In other words the wound is left to heal of its own accord.

The final postoperative aesthetic and functional result is remarkable. Dorsal elongated incisions of prepuce in paraphimoses and in hypospadiac surgery had a good healing by second invention. Automatic disruption of the prepuce in a case of nephrotic syndrome with elongated wounds also had excellent further alike healing. The absence of post-traumatic ingenerated cicatrix tissue is due to the rich prepuce blood vessels, in the absence of sutures and in usefulness of cicatrizing creams.

## Conclusion

Sutureless prepuceplasty is not an alternative method of circumcision in the treatment of phimosis, but a different technique in philosophy that improves the healing results, decrease the repairing time and the operation's cost and complications. The final postoperative results are the maintaining of functional properties and of natural prepuce morphology. The younger the children are, the better the healing results.

## Competing interests

The author(s) declare that they have no competing interests.

## Pre-publication history

The pre-publication history for this paper can be accessed here:


